# Psilocybin for clinical indications: A scoping review

**DOI:** 10.1177/02698811241269751

**Published:** 2024-08-13

**Authors:** Kim Madden, Breanne Flood, Darren Young Shing, Michael Ade-Conde, Imad Kashir, Melanie Mark, James MacKillop, Mohit Bhandari, Anthony Adili

**Affiliations:** 1 Research Institute of St. Joseph’s Healthcare Hamilton, Hamilton, ON, Canada; 2 Department of Surgery, McMaster University, Hamilton, ON, Canada; 3 Department of Health Research Methods, McMaster University, Hamilton, ON, Canada; 4 Michael G. DeGroote Center for Medicinal Cannabis Research, McMaster University, Hamilton, ON, Canada; 5 Faculty of Medicine, University of Ottawa, Ottawa, ON, Canada; 6 Royal College of Surgeons of Ireland, Dublin, Ireland; 7 Department of Psychiatry and Behavioral Neuroscience, McMaster University, Hamilton, ON, Canada; 8 Peter Boris Center for Addiction Research, McMaster University, Hamilton, ON, Canada

**Keywords:** Psilocybin, scoping review, pain management, psychedelic medicine

## Abstract

**Background:**

Psychedelic drugs have been of interest in medicine since the early 1950s. There has recently been a resurgence of interest in psychedelics.

**Aims:**

The objective of this study is to determine the extent of the available literature on psilocybin for medical indications including the designs used, study characteristics, indications studied, doses, and authors’ conclusions. We identify areas for further study where there are research gaps.

**Methods:**

We conducted a systematic scoping review of clinical indications for psilocybin, encompassing psychiatric and medical conditions. We systematically searched Medline and Embase using keywords related to psilocybin. We reviewed titles and texts in duplicate using Covidence software. We extracted data individually in duplicate using Covidence software and a senior reviewer resolved all author conflicts. We analyzed data descriptively.

**Results:**

We included 193 published and 80 ongoing studies. Thirty-seven percent of included studies were systematic reviews. Only 12% of included studies were randomized controlled trials. The median number of participants was 22 with a median of 18 participants who had taken psilocybin. Thirty-eight percent of studies reported at least one potential conflict of interest. The most common indication was depression (28%). Also commonly studied were substance use (14%), mental health in life-threatening illness (9%), headaches (6%), depression and anxiety (6%), obsessive-compulsive disorder (3%), and anxiety disorders (3%).

**Conclusions:**

Most studies involving the administration of psilocybin have small sample sizes and the most common focus has been psychiatric disorders. There is a need for high-quality randomized trials on psilocybin and to expand consideration to other promising indications, such as chronic pain.

## Introduction

Psychedelic drugs have been of interest in medicine and medical research since the early 1950s (Wollmann,1950). They became illegal for medical and recreational use in the United States in the 1970s (Johnson et al., 2018), effectively ending research into therapeutic applications of psychedelic drugs. There has recently been a resurgence of interest in clinical indications for psychedelic drugs.

Psilocybin is one such hallucinogenic drug that has been the subject of clinical research since the early 1960s (Rinkel et al., 1960). Psilocybin is a chemical compound from the tryptamine group and is derived from specific types of hallucinogenic mushrooms (Hofmann et al., 1958). It was first synthesized in 1958 and marketed as Indocybin by Sandoz Pharmaceuticals (Hofmann et al., 1958). Psilocybin is a pro-drug that is metabolized in the intestines and kidneys into its main active form, psilocin (Passie et al., 2002). Psilocin, as a 5H-HT_2A_ serotonin receptor agonist, can initiate a series of downstream effects in the brain. These effects encompass changes in neurotransmitter release, notably dopamine and glutamate, and can modulate perception, emotions, thoughts, and symptoms of anxiety or elation (Dinis-Oliveira, 2017; López-Giménez and González-Maeso, 2018). It is hypothesized that these actions may be effective in minimizing symptoms of mood disorders and substance use disorders, create a synergistic effect when used with psychotherapy or when taken in combination with other psychedelics, and potentially have other medical indications. Psilocybin is currently not legal for medical or recreational use in Canada (Health Canada, 2023) or the United States (Johnson et al., 2018), but it is possible there will be a change in the legal landscape, with growing interest in clinical applications. Recent approvals for limited psilocybin use in the United States have ignited substantial legislative action across 25 states, with 74 bills currently under consideration for psychedelic reform. These bills, predominantly centered on decriminalization, have seen a steady rise from 2019 to 2022, specifically targeting psilocybin (Siegel et al., 2023). Similarly, in Canada, some licensed healthcare professionals have successfully received exemptions to use therapeutic psilocybin for patients with life-threatening illnesses who have not had success with other therapies. In 2022, Health Canada clarified that psilocybin could be legally accessed through the Special Access Program (Health Canada, 2023) in special circumstances, or through approved clinical trials (Health Canada, 2022), based on the recent increased interest in therapeutic applications of psilocybin.

There has been a plethora of literature on psilocybin for medical purposes since the early 1960s. Although there have been systematic reviews on the topic, they tend to focus on a very specific subset of studies, or they focus on several psychedelic drugs. This study aims to provide a comprehensive overview of the available literature on psilocybin for any clinical indication, including the designs used, study characteristics, indications studied, and author’s conclusions. Through this scoping review, we seek to identify research gaps and areas for further study, to conduct high-quality randomized trials on promising clinical uses for psilocybin. This scoping review aims to provide insights for researchers and clinicians, guiding future investigations that will advance our understanding of psilocybin’s potential in clinical care.

## Methods

We conducted a systematic scoping review of any clinical indications for psilocybin. We systematically searched Medline and Embase in March 2023 using keywords related to psilocybin or psilocin. We kept the search very broad and used both keywords and MeSH/Emtree headings. We consulted a professional health sciences librarian (NB) when designing the search strategy. The full search strategy for each database is shown in Appendix A.

Studies were included if they evaluated psilocybin for any clinical indication. We set no date or language restrictions. Studies were excluded for the following reasons: recreational or non-clinical use, mechanistic or basic science studies, animal studies, narrative reviews, opinions or commentary articles, healthy volunteers only, protocols and methods papers, and studies where no full text was obtainable or where a translation could not be obtained (our team can translate French, Spanish, Mandarin, Dutch, and Polish).

We reviewed titles and then full texts individually in duplicate using Covidence software, with ties broken by a senior reviewer (KM). We designed a data extraction form specific to this study and pilot-tested it on a random sample of three included studies. We extracted data individually in duplicate using Covidence online software and a senior reviewer (KM) resolved all author conflicts.

We also systematically reviewed the clinicaltrials.gov trials registry for ongoing psilocybin trials broadly using the keyword “psilocybin” on March 22, 2023. We used the filters “active—not recruiting,” “enrolling by invitation,” “not yet recruiting,” and “recruiting” to identify ongoing trials. We excluded studies focusing only on healthy volunteers. We extracted status, indication/condition, study design, planned sample size, location, and expected or actual start and end dates from ongoing trials.

We analyzed data descriptively using frequencies and percentages for categorical data, graphically for the year of publication, and median with range for sample size, which was expected to be non-normally distributed.

## Results

### Study flow

**Figure 1.**
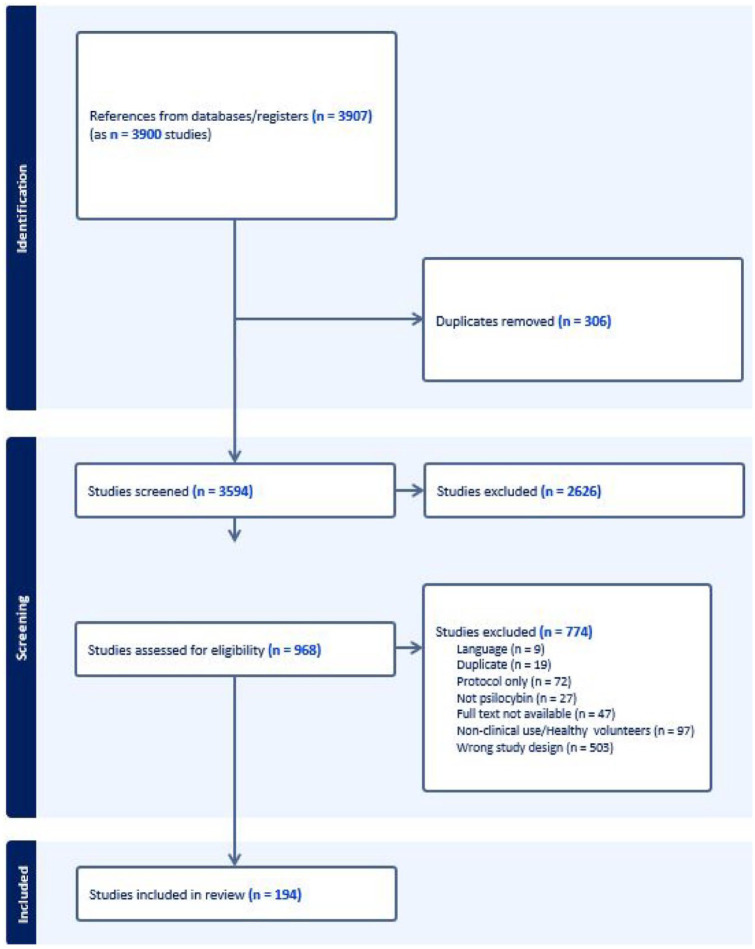
Study flow diagram.

After deduplicating published studies first using EndNote software and then using Covidence software, we reviewed 3907 studies for potential inclusion. After reviewing 968 full texts, we included 193 studies in this scoping review. Figure 1 shows the study flow diagram. We also included 80 ongoing studies from clinicaltrials.gov. A full list of included published and ongoing studies is shown found in Appendix B.

### Study characteristics

**Figure 2.**
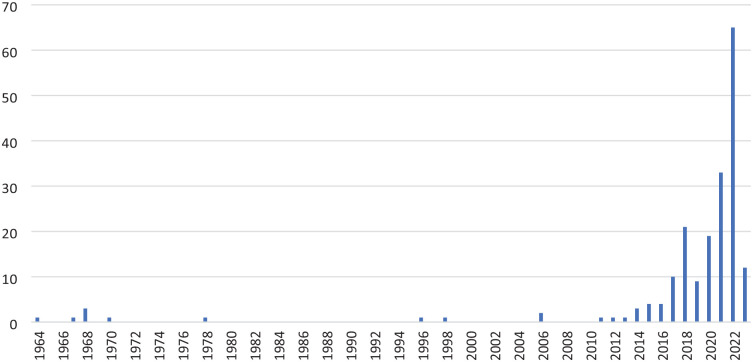
Number of publications by year.

The first study was published in 1964 and there were very few studies published annually until 2014–2017 when there was a sharp increase in the number of publications annually. More than half of all included studies were published in 2021–2023. Figure 2 shows the distribution of studies by year. The top four countries of publication were the United States (41.5%), the United Kingdom (15.5%), the Netherlands (5.2%), and Canada (4.7%) (Table 1). 37.3% of included studies were systematic reviews. There was a substantial number of non-randomized trials (e.g., healthy volunteers vs. clinical population) and single-arm trials (18.7%). Only 12.4% of included studies were randomized controlled trials (Table 1). Many of these included studies were conducted on duplicate populations (i.e., the same participants’ data was used in more than one publication). The 72 systematic reviews included anywhere from 1 to 86 studies on psilocybin. Of the 121 primary studies reporting their sample size, the median number of participants was 22 (range 1–484,732) with a median of 18 participants who had taken psilocybin (range 1–22,276). Of the 81 experimental studies where the investigators gave participants psilocybin (as opposed to naturalistic use), there was a total of 3480 participants and a median of 19 participants per study (range 1–405).

**Table 1.** Study characteristics.

Country	Number of studies	%
USA	80	41.5%
UK	30	15.6%
Netherlands	10	5.2%
Canada	9	4.7%
Australia	5	2.6%
Denmark	5	2.6%
Poland	5	2.6%
Portugal	5	2.6%
France	3	1.6%
Brazil	2	1.0%
Czechia	2	1.0%
Germany	2	1.0%
Mexico	2	1.0%
Norway	2	1.0%
Spain	2	1.0%
China	1	0.5%
Croatia	1	0.5%
Finland	1	0.5%
Ireland	1	0.5%
Israel	1	0.5%
Italy	1	0.5%
Japan	1	0.5%
New Zealand	1	0.5%
Sweden	1	0.5%
Switzerland	1	0.5%
Taiwan	1	0.5%
Uruguay	1	0.5%
Yemen	1	0.5%
Not reported	1	0.5%
Multiple	15	7.8%
Follow-up duration		
24 h or less	6	3.1%
1–2 weeks	5	2.6%
3–11 weeks	23	11.9%
12–51 weeks	19	9.8%
1–2 years	22	11.4%
More than 2 years	6	3.1%
Various	33	17.1%
Not reported/not applicable	79	40.1%
Study design		
Systematic review	72	37.1%
Non-randomized/single-arm trial	36	18.7%
Cross-sectional study/survey	25	13.0%
Randomized controlled trial	24	12.4%
Case report	13	6.7%
Qualitative research	9	4.7%
Cohort study	5	2.6%
Pooled analysis of 2+ studies	4	2.1%
Secondary analysis of RCT	3	1.5%
Case–control study	1	0.5%
Scoping review	1	0.5%
Funding source		
Not for profit	72	37.3%
None	39	20.2%
Individuals and non-profit	11	5.7%
For profit	9	4.7%
For profit and non-profit	4	2.1%
Individuals	1	0.5%
Not reported	57	29.5%
Conflicts of interest		
No relevant disclosures	74	38.3%
Potential conflicts declared	88	45.6%
Not reported	31	16.1%

### Study funding and conflicts

Most studies were funded by not-for-profit sources (37.3%) or were unfunded (20.2%), but a substantial number did not report their source of funding (29.5%). A total of 13 studies (6.7%) had at least one source of for-profit funding. 38.3% of studies reported no relevant conflicts of interest, 45.6% reported at least one potential conflict of interest (financial or intellectual), and 16.1% did not have a conflict of interest disclosure statement (Table 1).

### Clinical indications

The most common clinical indication was depression (28.0%). Also commonly studied were substance use disorders (14.5%), mental health in patients with life-threatening illness (8.8%), headaches (6.2%), depression and anxiety (5.7%), anxiety disorders (3.1%), obsessive-compulsive disorder (OCD; 3.1%), demoralization (1.6%), and bipolar disorder (1.6%). The other indications, each reported in only one study, included eating disorders, fibromyalgia, functional neurological disorders, neurosis, organic brain lesions, phantom limb pain, schizophrenia, autism, chronic pain, post-traumatic stress disorder (PTSD), suicidal ideation, irregular menstruation, and numerous studies reported on multiple indications (19.7%). A full list of clinical indications studied is shown in Table 2.

**Table 2.** Studies by indication.

Medical indication	Number of studies	%
Depression	54	28.0%
Substance use disorder	28	14.5%
Mental health in life-threatening illness	17	8.8%
Headaches	12	6.2%
Depression and anxiety	11	5.7%
Anxiety disorder	6	3.1%
OCD	6	3.1%
Eating disorders	4	2.1%
Demoralization	3	1.6%
Bipolar disorder	3	1.6%
Fibromyalgia	1	0.5%
Functional neurological disorders	1	0.5%
Neurosis	1	0.5%
Organic brain lesions	1	0.5%
Phantom limb pain	1	0.5%
Schizophrenia	1	0.5%
Autism	1	0.5%
Chronic pain	1	0.5%
PTSD	1	0.5%
Suicidal ideation	1	0.5%
Irregular menstruation	1	0.5%
Multiple indications	38	19.7%

### Intervention and control characteristics

In most cases, the manufacturer/supplier of psilocybin was not reported (87.1%). A hospital or research pharmacy supplied the drug in 2.1% of studies. Four different companies supplied the drug in several studies, two studies used homegrown psilocybin mushrooms, and in one study the mushrooms were purchased from the local community. Psychotherapy was explicitly used in 43.8% of studies as part of psychedelic-assisted psychotherapy. In 48.2% of studies, there was no control group. 22.3% of studies used various controls, and the control was unclear in 8.3% of studies. The five most common controls used were waitlist control (3.6%), diphenhydramine, healthy volunteers, niacin, and unspecified placebo (2.6% each). Primary outcomes were assessed through various methods. 38.9% of studies used self-report questionnaires, 3.6% of studies used neuroimaging, and 2.1% used laboratory samples. Other assessments such as performance-based assessments, clinician reports, and monitoring adverse events were each used in less than 1% of studies. The authors’ conclusions on the efficacy or safety of psilocybin were positive or promising in 59.1% of studies, mixed (e.g., benefits and harms, effective for some, more research needed) in 11.4% of studies, negative (e.g., ineffective, harmful) in 2.1% of studies, and 27.5% of studies had a different conclusion or had no conclusions regarding efficacy or safety. Of the 81 experimental studies, the author’s conclusions on the efficacy or safety of psilocybin were positive or promising in 55.6% of studies, mixed (e.g., benefits and harms, effective for some, more research needed) in 24.7% of studies, negative (e.g., ineffective, harmful) in 1.2% of studies, and 30.9% of studies had a different conclusion or there were no conclusions about efficacy or safety. The full intervention and control characteristics are shown in Table 3.

**Table 3.** Intervention and control characteristics.

Manufacturer/source		
Not reported	168	87.1%
Compass pathways	9	4.7%
Hospital/university research pharmacy	4	2.1%
THC pharm	4	2.1%
Usona institute	3	1.6%
Sandoz	2	1.0%
Homegrown	2	1.0%
Purchased from the local community	1	0.5%
Control group		
No control	93	48.2%
Various	43	22.3%
Unclear/not reported	16	8.3%
Waitlist control	7	3.6%
Diphenhydramine	5	2.6%
Healthy volunteers	5	2.6%
Niacin	5	2.6%
Placebo—unspecified	5	2.6%
Escitalopram	3	1.6%
High dose vs. low dose	3	1.6%
Very low-dose psilocybin	3	1.6%
Non-users of psychedelics	2	1.0%
LSD	1	0.5%
Microcrystalline cellulose	1	0.5%
Nicotine patch	1	0.5%
Psychotherapy explicitly used		
Yes	84	43.8%
Primary outcome assessment		
Self-report/questionnaire	75	38.9%
Neuroimaging	7	3.6%
Laboratory samples	4	2.1%
Performance-based assessment	1	0.5%
Clinician reported	1	0.5%
Adverse events	1	0.5%
Unclear	1	0.5%
Not reported	4	2.1%
N/A	99	51.3%
Author’s conclusions		
Positive conclusions (e.g., effective, promising, patient supportive)	114	59.1%
Mixed conclusions (e.g., benefits and harms, effective for some, more research needed)	22	11.4%
Negative (e.g., ineffective, harmful)	4	2.1%
Other/not an effective conclusion	53	27.5%

### Ongoing studies

There were 100 ongoing studies on psilocybin listed on clinicaltrials.gov, of which we excluded 20 for not having a clinical indication (i.e., healthy volunteers only). Therefore, we included 80 ongoing studies: 42.5% of the studies are recruiting, 35.0% are not yet recruiting, 20.0% are active but not yet recruiting, and 2.5% are recruiting by invitation. 67.5% of studies are being conducted in the United States, 8.8% in Canada, and the remainder are in Europe and New Zealand, or several countries. Depression (36.3%), substance use disorder (17.5%), and psychological distress in terminal illness or cancer (8.8%) are the three most common indications studied. 42.5% of ongoing studies are randomized parallel assignment trials, 40.0% are single-arm trials, 12.5% are crossover randomized trials, 3.8% are non-randomized comparative trials, and 1 (1.3%) is a prospective cohort study. The median number of participants planned to be in the study is 30 (range 10–568). Full ongoing study characteristics are shown in Table 4.

**Table 4.** Ongoing study characteristics.

Status	Number of ongoing studies	%
Recruiting	34	42.5%
Not yet recruiting	28	35.0%
Active, not recruiting	16	20.0%
Enrolling by invitation	2	2.5%
Country		
USA	54	67.5%
Canada	7	8.8%
United Kingdom	6	7.5%
Denmark	2	2.5%
Czechia	1	1.3%
Germany	1	1.3%
New Zealand	1	1.3%
Sweden	1	1.3%
Switzerland	1	1.3%
Multiple countries	5	6.3%
Indication		
Depression	29	36.3%
Substance use disorder	14	17.5%
Psychological distress in terminal illness/cancer	7	8.8%
Obsessive-compulsive disorder	5	6.3%
Eating disorder	4	5.0%
Headache/migraine	4	5.0%
Post-traumatic stress disorder	3	3.8%
Fibromyalgia	3	3.8%
Demoralization	2	2.5%
Autism	1	1.3%
Bipolar disorder	1	1.4%
Burnout	1	1.3%
Chronic low-back pain	1	1.3%
Neurological disorders	1	1.3%
Perception disorders	1	1.3%
Phantom limb pain	1	1.3%
Chronic Lyme disease	1	1.3%
Nervous system trauma	1	1.3%
Study design		
Parallel randomized	34	42.5%
Single-arm trial	32	40.0%
Crossover randomized	10	12.5%
Non-randomized	3	3.8%
Prospective cohort	1	1.3%
Start year		
2008	1	1.3%
2015	1	1.3%
2016	1	1.3%
2017	1	1.3%
2018	2	2.7%
2019	4	5.0%
2020	3	3.8%
2021	14	17.5%
2022	24	30.0%
2023*	29	36.3%
Planned completion year		
2022	1	1.3%
2023	31	38.8%
2024	28	35.0%
2025	14	17.5%
2026	4	5.0%
2027	2	2.7%

* May be a planned start time.

## Discussion

Although there is a substantial body of literature available on psilocybin for clinical indications spanning more than 6 decades, there are relatively few primary studies and even fewer high-quality randomized controlled trials. Many of the included studies were performed decades ago and many of the recent trials were conducted in duplicate populations (e.g., substudies of the same primary trial), indicating a need to conduct new primary studies in a variety of populations. The clinicaltrials.gov registrations indicate that many new primary studies are being conducted, but they are also typically small and non-randomized. Clinical trials, especially large randomized controlled trials conducted in diverse patient populations, are imperative for providing robust evidence on psilocybin’s effects, minimizing bias, enhancing the generalizability of results, and are required for regulatory submissions for approval of new drugs.

Most of the included studies were performed in the field of psychiatric disorders. There were 12 completed studies on headaches, one on unspecified chronic pain, and one on phantom limb pain that may indicate a potential role of psilocybin in chronic pain. In addition, there are 10 ongoing studies listed in clinicaltrials.gov on pain conditions including phantom limb pain, low back pain, fibromyalgia, and headache/migraine, indicating growing interest in areas outside mental health.

There have been previous scoping reviews and systematic reviews on psilocybin, but they are typically focused on a narrow indication or set of indications. A previous scoping review of psilocybin-assisted psychotherapy for cancer patients concluded that psilocybin is promising but that the field is in its early stages (Lehto et al., 2021). Several systematic reviews on anxiety and/or depression have similarly concluded that psilocybin appears to be safe and effective, but that the existing studies are small and have important limitations that larger RCTs could address (Sarris et al., 2022; Vargas et al., 2020; Yu et al., 2021, 2022). A systematic review of the safety of psilocybin concluded that it appears to be safe, given that the reported adverse effects are typically manageable. However, they also caution that larger trials are needed to confirm the safety in larger samples and to help identify rare complications that may occur (Rossi et al., 2022). Although the vast majority of systematic reviews focused on mental health conditions, there was one systematic review that evaluated psilocybin for cluster headaches (Rusanen et al., 2022). This review was based entirely on survey studies, but it found that patients consistently report positive effects (e.g., reduced intensity or frequency of headaches) of psilocybin for headaches and concluded that more research is needed in this area.

This scoping review has several strengths including the broad and systematic search criteria and inclusion of studies from the 1960s until the present. A limitation of this approach is that older studies are more difficult to access, so we cannot be sure we have included all studies from several decades ago. We reviewed each article and extracted data independently and in duplicate, minimizing data entry errors. For ongoing trials, we only assessed clinicaltrials.gov, which disproportionately includes trials from North America. There may be other ongoing trials from different regions that we did not describe. In addition, clinicaltrials.gov infrequently contains study designs other than clinical trials, so there may be other ongoing studies of different designs that are not registered notably systematic reviews.

In conclusion, we have identified that there is a large body of psilocybin research spanning several decades and showing a substantial increase in recent years. However, much of the published research is preliminary. Although more RCTs are being conducted, they remain small in sample size and narrow in their indications. The most studied indications for psilocybin use are psychiatric disorders. There is an opportunity to expand to other potential indications where psilocybin may be of medical use, like chronic pain.

## Supplemental Material

sj-docx-1-jop-10.1177_02698811241269751 – Supplemental material for Psilocybin for clinical indications: A scoping review Supplemental material, sj-docx-1-jop-10.1177_02698811241269751 for Psilocybin for clinical indications: A scoping review by Kim Madden, Breanne Flood, Darren Young Shing, Michael Ade-Conde, Imad Kashir, Melanie Mark, James MacKillop, Mohit Bhandari and Anthony Adili in Journal of Psychopharmacology
