# Supplementary material for: Psilocybin for clinical indications: A scoping review
Source: J Psychopharmacol. 2024 Aug 13;38(10):839–45. doi: 10.1177/02698811241269751 (PMC11481402; doi:10.1177/02698811241269751)
Supplement: sj-docx-1-jop-10.1177_02698811241269751 – Supplemental material for Psilocybin for clinical indications: A scoping review [file sj-docx-1-jop-10.1177_02698811241269751.docx]

**Appendix A: Search Strategies**

**Medline Ovid – 8 March 2023**

| 1 | psilocybin.mp. or exp Psilocybin/ | 1572 |
| --- | --- | --- |
| 2 | psilocin.mp. | 261 |
| 3 | hallucinogenic mushroom*.mp. | 106 |
| 4 | psychedelic mushroom*.mp. | 13 |
| 5 | Psilocybe/ or magic mushroom*.mp. | 160 |
| 6 | 1 or 2 or 3 or 4 or 5 | 1726 |

**Embase Ovid – 8 March 2023**

| 1 | psilocybin.mp. or exp psilocybine/ | 2537 |
| --- | --- | --- |
| 2 | psilocin.mp. or exp psilocin/ | 573 |
| 3 | exp hallucinogenic fungus/ or hallucinogenic mushroom*.mp. | 256 |
| 4 | psychedelic mushroom*.mp. | 19 |
| 5 | magic mushroom*.mp. or exp Psilocybe/ | 303 |
| 6 | 1 or 2 or 3 or 4 or 5 | 2930 |

**Psycinfo Ovid – 8 March 2023**

| 1 | psilocybin.mp. or exp Psilocybin/ | 779 |
| --- | --- | --- |
| 2 | psilocin.mp. | 50 |
| 3 | hallucinogenic mushroom*.mp. | 26 |
| 4 | psychedelic mushroom*.mp. | 10 |
| 5 | magic mushroom*.mp. | 36 |
| 6 | psilocybe.mp. | 32 |
| 7 | 1 or 2 or 3 or 4 or 5 or 6 | 836 |

**Central Ovid – 8 March 2023**

| 1 | psilocybin.mp. or exp Psilocybin/ | 284 |
| --- | --- | --- |
| 2 | psilocin.mp. | 21 |
| 3 | hallucinogenic mushroom*.mp. | 0 |
| 4 | 1 or 2 or 3 | 288 |

**Appendix B – List of included studies**

**Published studies (n=193)**

1. Aaronson S, Miller T, Rudow S, Forbes M, Suppes T. P388. Open Label Psilocybin Administration in Severely Treatment Resistant Depression. Biological Psychiatry. 2022;91(9 Supplement):S244.
2. Aaronson S, Suppes T, Miller T, Rudow S, Forbes M, Shoultz A. An Open Label Study of COMP360 (Synthetic Psilocybin) in Bipolar Type II Depression. Neuropsychopharmacology. 2022;47(Supplement 1):197-8.
3. Aday JS, Mitzkovitz CM, Bloesch EK, Davoli CC, Davis AK. Long-term effects of psychedelic drugs: A systematic review. Neuroscience & Biobehavioral Reviews. 2020;113:179-89.
4. Agin-Liebes G. The role of self-compassion in psilocybin-assisted motivational enhancement therapy to treat alcohol dependence: A randomized controlled trial. Dissertation Abstracts International: Section B: The Sciences and Engineering. 2021;82(9-B):No Pagination Specified.
5. Agin-Liebes GI, Malone T, Yalch MM, Mennenga SE, Ponte KL, Guss J, et al. Long-term follow-up of psilocybin-assisted psychotherapy for psychiatric and existential distress in patients with life-threatening cancer. Journal of Psychopharmacology. 2020;34(2):155-66.
6. Agrawal M, Thambi PM, Shnayder S. The safety and efficacy of psilocybin therapy in patients with cancer and major depressive disorder. Journal of Clinical Oncology Conference: Annual Meeting of the American Society of Clinical Oncology, ASCO. 2022;40(16 Supplement 1).
7. Al-Naggar RA, Alshaikhli H, Erlam G. Effectiveness of psilocybin on depression: A qualitative study. Electronic Journal of General Medicine. 2021;18(3) (no pagination).
8. Alfafar M, Farinha R, Pontes F. P.0620 Psilocybin as a treatment of addictions: a review of the literature. European Neuropsychopharmacology. 2021;53(Supplement 1):S455-S6.
9. Alnaes R. Therapeutic Application of the Change in Consciousness Produced by Psycholytica (Lsd, Psilocybin, Etc.). The Psychedelic Experience in the Treatement of Neurosis. Acta Psychiatrica Scandinavica. 1964;39(S180):397-409.
10. Altman BR, Earleywine M, De Leo J. Exploring the Credibility of Psilocybin-assisted Therapy and Cognitive-behavioral Therapy for Depression. Journal of Psychoactive Drugs. 2022:1-9.
11. Amegadzie S, Mennenga S, Podrebarac S, Duane H, Ross S, Bogenschutz M. Psilocybin-assisted treatment for alcohol use disorder: A clinical perspective. American Journal on Addictions. 2018;27(4):317.
12. Andersen KAA, Carhart-Harris R, Nutt DJ, Erritzoe D. Therapeutic effects of classic serotonergic psychedelics: A systematic review of modern-era clinical studies. Acta Psychiatr Scand. 2021;143(2):101-18.
13. Anderson BT, Danforth A, Daroff PR, Stauffer C, Ekman E, Agin-Liebes G, et al. Psilocybin-assisted group therapy for demoralized older long-term AIDS survivor men: An open-label safety and feasibility pilot study. EClinicalMedicine. 2020;27:100538.
14. Andersson M, Persson M, Kjellgren A. Psychoactive substances as a last resort-a qualitative study of self-treatment of migraine and cluster headaches. Harm Reduction Journal. 2017;14(1):60.
15. Barba T, Buehler S, Kettner H, Radu C, Cunha BG, Nutt DJ, et al. Effects of psilocybin versus escitalopram on rumination and thought suppression in depression. BJPsych Open. 2022;8(5):e163.
16. Barrett F. In patients with major depressive disorder, psilocybin administration is associated with reduced amygdala response to negative affective stimuli and normalization of cortical glutamate one week after psilocybin, and improved cognitive flexibility one and four weeks after psilocybin. Neuropsychopharmacology. 2019;44(Supplement 1):76-7.
17. Begola MJ, Schillerstrom JE. Hallucinogens and Their Therapeutic Use: A Literature Review. Journal of Psychiatric Practice. 2019;25(5):334-46.
18. Belser AB, Agin-Liebes G, Swift T, Terrana S, Devenot N, Friedman HL, et al. Patient experiences of psilocybin-assisted psychotherapy: An interpretative phenomenological analysis. Journal of Humanistic Psychology. 2017;57(4):354-88.
19. Bender D, Hellerstein DJ. Assessing the risk-benefit profile of classical psychedelics: a clinical review of second-wave psychedelic research. Psychopharmacology. 2022;239(6):1907-32.
20. Benville J, Agin-Liebes G, Roberts DE, Lo S, Ghazal L, Franco-Corso SJ, et al. Effects of Psilocybin on Suicidal Ideation in Patients With Life-Threatening Cancer. Biological Psychiatry. 2021;89(9 Supplement):S235-S6.
21. Bogadi M, Kastelan S. A potential effect of psilocybin on anxiety in neurotic personality structures in adolescents. Croatian Medical Journal. 2021;62(5):528-30.
22. Bogenschutz MP. Neuropsychopharmacology. 2012;1):S15-S6.
23. Bogenschutz MP, Forcehimes AA, Pommy JA, Wilcox CE, Barbosa PC, Strassman RJ. Psilocybin-assisted treatment for alcohol dependence: a proof-of-concept study. Journal of Psychopharmacology. 2015;29(3):289-99.
24. Bogenschutz MP, Podrebarac SK, Duane JH, Amegadzie SS, Malone TC, Owens LT, et al. Clinical Interpretations of Patient Experience in a Trial of Psilocybin-Assisted Psychotherapy for Alcohol Use Disorder. Frontiers in Pharmacology. 2018;9:100.
25. Bogenschutz MP, Ross S, Bhatt S, Baron T, Forcehimes AA, Laska E, et al. Percentage of Heavy Drinking Days Following Psilocybin-Assisted Psychotherapy vs Placebo in the Treatment of Adult Patients With Alcohol Use Disorder: a Randomized Clinical Trial. JAMA psychiatry. 2022;79(10):953‐62.
26. Breeksema JJ, Kuin BW, Kamphuis J, van den Brink W, Vermetten E, Schoevers RA. Adverse events in clinical treatments with serotonergic psychedelics and MDMA: A mixed-methods systematic review. Journal of Psychopharmacology. 2022;36(10):1100-17.
27. Breeksema JJ, Niemeijer AR, Krediet E, Vermetten E, Schoevers RA. Psychedelic Treatments for Psychiatric Disorders: A Systematic Review and Thematic Synthesis of Patient Experiences in Qualitative Studies. CNS Drugs. 2020;34(9):925-46.
28. Butler M, Seynaeve M, Nicholson TR, Pick S, Kanaan RA, Lees A, et al. Psychedelic treatment of functional neurological disorder: a systematic review. Therapeutic Advances in Psychopharmacology. 2020;10:2045125320912125.
29. Calleja-Conde J, Morales-Garcia JA, Echeverry-Alzate V, Buhler KM, Gine E, Lopez-Moreno JA. Classic psychedelics and alcohol use disorders: A systematic review of human and animal studies. Addiction Biology. 2022;27(6):e13229.
30. Cao B, Zhu J, Zuckerman H, Rosenblat JD, Brietzke E, Pan Z, et al. Pharmacological interventions targeting anhedonia in patients with major depressive disorder: A systematic review. Progress in Neuro-Psychopharmacology & Biological Psychiatry. 2019;92:109-17.
31. Capaldi JM, Shabanian J, Finster LB, Asher A, Wertheimer JC, Zebrack BJ, et al. Post-traumatic stress symptoms, post-traumatic stress disorder, and post-traumatic growth among cancer survivors: a systematic scoping review of interventions. Health psychol. 2023:1-34.
32. Carhart-Harris R. Results: Of a multi-modal neuroimaging study of LSD and a psilocybin for treatment-resistant depression clinical trial. Neuropsychopharmacology. 2015;1):S91-S2.
33. Carhart-Harris R, Giribaldi B, Watts R, Baker-Jones M, Murphy-Beiner A, Murphy R, et al. Trial of Psilocybin versus Escitalopram for Depression. New England Journal of Medicine. 2021;384(15):1402-11.
34. Carhart-Harris RL, Bolstridge M, Day CMJ, Rucker J, Watts R, Erritzoe DE, et al. Psilocybin with psychological support for treatment-resistant depression: six-month follow-up. Psychopharmacology. 2017.
35. Carhart-Harris RL, Roseman L, Bolstridge M, Demetriou L, Pannekoek JN, Wall MB, et al. Psilocybin for treatment-resistant depression: fMRI-measured brain mechanisms. Scientific Reports. 2017;7(1):13187.
36. Carrillo F, Sigman M, Fernandez Slezak D, Ashton P, Fitzgerald L, Stroud J, et al. Natural speech algorithm applied to baseline interview data can predict which patients will respond to psilocybin for treatment-resistant depression. Journal of Affective Disorders. 2018;230:84-6.
37. Castro Santos H, Gama Marques J. What is the clinical evidence on psilocybin for the treatment of psychiatric disorders? A systematic review. Porto Biomed J. 2021;6(1):e128.
38. Clifton JM, Belcher AM, Greenblatt AD, Welsh CM, Cole TO, Davis AK. Psilocybin use patterns and perception of risk among a cohort of Black individuals with Opioid Use Disorder. Journal of Psychedelic Studies. 2022;6(2):80-7.
39. Corrigan K, Haran M, McCandliss C, McManus R, Cleary S, Trant R, et al. Psychedelic perceptions: mental health service user attitudes to psilocybin therapy. Irish Journal of Medical Science. 2022;191(3):1385-97.
40. Crowe M, Manuel J, Carlyle D, Lacey C. Experiences of psilocybin treatment for clinical conditions: A qualitative meta-synthesis. Int J Ment Health Nurs. 2023;13:13.
41. Davis AK, Barrett FS, May DG, Cosimano MP, Sepeda ND, Johnson MW, et al. Effects of Psilocybin-Assisted Therapy on Major Depressive Disorder: A Randomized Clinical Trial. JAMA Psychiatry. 2021;78(5):481-9.
42. Daws RE, Timmermann C, Giribaldi B, Sexton JD, Wall MB, Erritzoe D, et al. Increased global integration in the brain after psilocybin therapy for depression. Nature Medicine. 2022;28(4):844-51.
43. de Coo IF, Naber WC, Wilbrink LA, Haan J, Ferrari MD, Fronczek R. Increased use of illicit drugs in a Dutch cluster headache population. Cephalalgia. 2019;39(5):626-34.
44. DellaCrosse M, Pleet M, Morton E, Ashtari A, Sakai K, Woolley J, et al. "A sense of the bigger picture:" A qualitative analysis of follow-up interviews with people with bipolar disorder who self-reported psilocybin use. PLoS ONE [Electronic Resource]. 2022;17(12):e0279073.
45. Di Lorenzo C, Coppola G, Di Lorenzo G, Bracaglia M, Rossi P, Pierelli F. The use of illicit drugs as self-medication in the treatment of cluster headache: Results from an Italian online survey. Cephalalgia. 2016;36(2):194-8.
46. Dos Santos RG, Bouso JC, Alcazar-Corcoles MA, Hallak JEC. Efficacy, tolerability, and safety of serotonergic psychedelics for the management of mood, anxiety, and substance-use disorders: a systematic review of systematic reviews. Expert Review of Clinical Pharmacology. 2018;11(9):889-902.
47. Dos Santos RG, Osorio FL, Crippa JA, Riba J, Zuardi AW, Hallak JE. Antidepressive, anxiolytic, and antiaddictive effects of ayahuasca, psilocybin and lysergic acid diethylamide (LSD): a systematic review of clinical trials published in the last 25 years. Therapeutic Advances in Psychopharmacology. 2016;6(3):193-213.
48. Dubansky B, Vyhnankova M, Setlik L. [Concurrent appearance of neurologic syndromes and of altered proprioceptive perception after psilocybine in patients with organic brain damage]. Ceskoslovenska Neurologie. 1968;31(6):394-9.
49. Earleywine M, Low F, Altman BR, De Leo J. How Important Is a Guide Who Has Taken Psilocybin in Psilocybin-Assisted Therapy for Depression? Journal of Psychoactive Drugs. 2022:1-11.
50. Ehrmann K, Allen JJB, Moreno FA. Psilocybin for the Treatment of Obsessive-Compulsive Disorders. Current Topics in Behavioral Neurosciences. 2021;17:17.
51. Erritzoe D, Roseman L, Nour M, MacLean K, Kaelen M, Nutt D, et al. Effects of psilocybin therapy on personality structure. Acta Psychiatrica Scandinavica. 2018;138(5):368-78.
52. Felsch CL, Kuypers KPC. Don't be afraid, try to meditate- potential effects on neural activity and connectivity of psilocybin-assisted mindfulness-based intervention for social anxiety disorder: A systematic review. Neuroscience & Biobehavioral Reviews. 2022;139:104724.
53. Fisher G. The psycholytic treatment of a childhood schizophrenic girl. International Journal of Social Psychiatry. 1970;16(2):112-30.
54. Freeman E, Adair M, Beeler D, Casper R, Herman MP, Reeves D, et al. Patient-identified burden and unmet needs in patients with cluster headache: An evidence-based qualitative literature review. Cephalalgia Reports. 2022;5(no pagination).
55. Galvao-Coelho NL, Marx W, Gonzalez M, Sinclair J, de Manincor M, Perkins D, et al. Classic serotonergic psychedelics for mood and depressive symptoms: a meta-analysis of mood disorder patients and healthy participants. Psychopharmacology. 2021;238(2):341-54.
56. Garay RP, Zarate CA, Charpeaud T, Citrome L, Correll CU, Hameg A, et al. Investigational drugs in recent clinical trials for treatment-resistant depression. Expert Review of Neurotherapeutics. 2017;17(6):593-609.
57. Garcia-Romeu A, Barrett FS, Carbonaro TM, Johnson MW, Griffiths RR. Optimal dosing for psilocybin pharmacotherapy: Considering weight-adjusted and fixed dosing approaches. Journal of Psychopharmacology. 2021;35(4):353-61.
58. Garcia-Romeu A, Griffiths RR, Johnson MW. Psilocybin-occasioned mystical experiences in the treatment of tobacco addiction. Current Drug Abuse Reviews. 2014;7(3):157-64.
59. Garcia-Romeu A, Griffiths RR, Johnson MW. Psychedelic-associated addiction remission: An online survey. Drug and Alcohol Dependence. 2017;171:e69.
60. Garcia-Romeu AP, Griffiths RR, Johnson MW. Psychedelic-facilitated smoking cessation: An online survey. Drug and Alcohol Dependence. 2015;146:e120.
61. Gattuso JJ, Perkins D, Ruffell S, Lawrence AJ, Hoyer D, Jacobson LH, et al. Default Mode Network Modulation by Psychedelics: A Systematic Review. International Journal of Neuropsychopharmacology. 2022;22:22.
62. Geert-Jorgensen E. Further observations regarding hallucinogenic treatment. Acta Psychiatrica Scandinavica, Supplementum. 1968;203:195-200.
63. Gill H, Puramat P, Patel P, Gill B, Marks CA, Rodrigues NB, et al. The Effects of Psilocybin in Adults with Major Depressive Disorder and the General Population: Findings from Neuroimaging Studies. Psychiatry Research. 2022;313:114577.
64. Glynos NG, Pierce J, Davis AK, McAfee J, Boehnke KF. Knowledge, Perceptions, and Use of Psychedelics among Individuals with Fibromyalgia. Journal of Psychoactive Drugs. 2022:1-12.
65. Goldberg SB, Pace BT, Nicholas CR, Raison CL, Hutson PR. The experimental effects of psilocybin on symptoms of anxiety and depression: A meta-analysis. Psychiatry Research. 2020;284:112749.
66. Goldberg SB, Shechet B, Nicholas CR, Ng CW, Deole G, Chen Z, et al. Post-acute psychological effects of classical serotonergic psychedelics: a systematic review and meta-analysis. Psychological Medicine. 2020;50(16):2655-66.
67. Gomez-Busto FJ, Ortiz MI. Virtual reality and psychedelics for the treatment of psychiatric disease: A systematic literature review. Clinical Neuropsychiatry. 2020;17(6):365-80.
68. Goodwin G, Feifel D, Hellerstein DJ, Kelly JR, Marwood L, Mistry S, et al. Dose-Dependent Acute Subjective Psychedelic Effects Following COMP360 Psilocybin Across Three Clinical Studies and its Relationship to Therapeutic Response. Neuropsychopharmacology. 2022;47(Supplement 1):201.
69. Goodwin G, Simmons H, Marwood L, Mistry S, Schlosser D, Tsai J, et al. Predicting Depression Outcomes Through the Influence of Therapeutic Alliance and the Psychedelic Experience Using Path Modelling in a Phase IIb Randomized Controlled Trial of COMP360 Psilocybin Therapy. Neuropsychopharmacology. 2022;47(Supplement 1):200.
70. Goodwin GM, Aaronson ST, Alvarez O, Arden PC, Baker A, Bennett JC, et al. Single-Dose Psilocybin for a Treatment-Resistant Episode of Major Depression. New England Journal of Medicine. 2022;387(18):1637-48.
71. Griffiths R, Barrett F, Darrick M, Johnson M, Mary C, Patrick F, et al. Psilocybin-assisted treatment of major depressive disorder: Results from a randomized trial. Neuropsychopharmacology. 2019;44(Supplement 1):439.
72. Griffiths RR, Johnson MW, Carducci MA, Umbricht A, Richards WA, Richards BD, et al. Psilocybin produces substantial and sustained decreases in depression and anxiety in patients with life-threatening cancer: A randomized double-blind trial. Journal of Psychopharmacology. 2016;30(12):1181-97.
73. Grob CS, Danforth AL, Chopra GS, Hagerty M, McKay CR, Halberstad AL, et al. Pilot study of psilocybin treatment for anxiety in patients with advanced-stage cancer. Archives of General Psychiatry. 2011;68(1):71-8.
74. Gukasyan N. 19.3 Psilocybin-Assisted Therapy for MDD: Current Evidence and Clinical Considerations. Journal of the American Academy of Child and Adolescent Psychiatry. 2022;61(10 Supplement):S305-S6.
75. Gukasyan N, Davis AK, Barrett FS, Cosimano MP, Sepeda ND, Johnson MW, et al. Efficacy and safety of psilocybin-assisted treatment for major depressive disorder: Prospective 12-month follow-up. Journal of Psychopharmacology. 2022;36(2):151-8.
76. Gukasyan N, Narayan SK. Menstrual Changes and Reversal of Amenorrhea Induced by Classic Psychedelics: A Case Series. Journal of Psychoactive Drugs. 2022.
77. Hansen LC, Gaul C, Pogatzki-Zahn E, Baron R, Gierthmuhlen J. Do doctors treat themselves differently than their patients? Study on the self-treatment of migraine among German neurologists and pain specialists. Cephalalgia. 2020;40(8):788-96.
78. Harding F, Seynaeve M, Keeler J, Himmerich H, Treasure J, Kan C. Perspectives on psychedelic treatment and research in eating disorders: A web-based questionnaire study of people with eating disorders. NeuroSignals. 2021;20(3):551-60.
79. Hendricks P. Psilocybin in the Treatment of Cocaine Use Disorder. Neuropsychopharmacology. 2022;47(Supplement 1):34.
80. Heuschkel K, Kuypers KP. Depression, mindfulness, and psilocybin: Possible complementary effects of mindfulness meditation and psilocybin in the treatment of depression. A review. Frontiers in Psychiatry Vol 11 2020, ArtID 224. 2020;11.
81. Hodge AT, Sukpraprut-Braaten S, Narlesky M, Strayhan RC. The Use of Psilocybin in the Treatment of Psychiatric Disorders with Attention to Relative Safety Profile: A Systematic Review. Journal of Psychoactive Drugs. 2022:1-11.
82. Horton DM, Morrison B, Schmidt J. Systematized Review of Psychotherapeutic Components of Psilocybin-Assisted Psychotherapy. Am J Psychother. 2021;74(4):140-9.
83. Hutten N, Mason NL, Dolder PC, Kuypers KPC. Self-Rated Effectiveness of Microdosing With Psychedelics for Mental and Physical Health Problems Among Microdosers. Frontiers in psychiatry Frontiers Research Foundation. 2019;10:672.
84. rizarry R, Winczura A, Dimassi O, Dhillon N, Minhas A, Larice J. Psilocybin as a Treatment for Psychiatric Illness: A Meta-Analysis. Cureus. 2022;14(11):e31796.
85. Johnson M. Psilocybin in the Treatment of Tobacco Use Disorder. Neuropsychopharmacology. 2022;47(Supplement 1):34.
86. Johnson MW, Garcia-Romeu A, Cosimano MP, Griffiths RR. Pilot study of the 5-HT2AR agonist psilocybin in the treatment of tobacco addiction. J Psychopharmacol. 2014;28(11):983-92.
87. Johnson MW, Garcia-Romeu A, Griffiths RR. Long-term follow-up of psilocybin-facilitated smoking cessation. Am J Drug Alcohol Abuse. 2017;43(1):55-60.
88. Johnson MW, Garcia-Romeu A, Johnson PS, Griffiths RR. An online survey of tobacco smoking cessation associated with naturalistic psychedelic use. Journal of Psychopharmacology. 2017;31(7):841-50.
89. Johnson S, Black QC. Classic psychedelics as a psychotherapeutic aid in the treatment of stimulant use disorder: A case report. International Journal of Mental Health and Addiction. 2020:No Pagination Specified.
90. Jones G, Arias D, Nock M. Associations between MDMA/ecstasy, classic psychedelics, and suicidal thoughts and behaviors in a sample of U.S. adolescents. Scientific Reports. 2022;12(1):21927.
91. Jones G, Lipson J, Nock MK. Associations between classic psychedelics and nicotine dependence in a nationally representative sample. Scientific Reports. 2022;12(1):10578.
92. Jones GM, Nock MK. Exploring protective associations between the use of classic psychedelics and cocaine use disorder: a population-based survey study. Scientific Reports. 2022;12(1):2574.
93. Jones GM, Nock MK. Race and ethnicity moderate the associations between lifetime psychedelic use (MDMA and psilocybin) and psychological distress and suicidality. Scientific Reports. 2022;12(1):16976.
94. Jungaberle H, Thal S, Zeuch A, Rougemont-Bucking A, von Heyden M, Aicher H, et al. Neuropharmacology. 2018;142:179-99.
95. Kaelen M, Giribaldi B, Raine J, Evans L, Timmerman C, Rodriguez N, et al. The hidden therapist: evidence for a central role of music in psychedelic therapy. Psychopharmacology. 2018;235(2):505-19.
96. Kelmendi B, Kichuk SA, DePalmer G, Maloney G, Ching THW, Belser A, et al. Single-dose psilocybin for treatment-resistant obsessive-compulsive disorder: A case report. Heliyon. 2022;8(12):e12135.
97. Kisely S, Connor M, Somogyi AA, Siskind D. A systematic literature review and meta-analysis of the effect of psilocybin and methylenedioxymethamphetamine on mental, behavioural or developmental disorders. Australian & New Zealand Journal of Psychiatry. 2022:48674221083868.
98. Knatz Peck S, Shao S, Murray S, Kaye W. P450. Pilot Study Evaluation of Psilocybin Therapy for Anorexia Nervosa: Safety, Acceptability, and Preliminary Efficacy. Biological Psychiatry. 2022;91(9 Supplement):S270.
99. Ko K, Knight G, Rucker JJ, Cleare AJ. Psychedelics, Mystical Experience, and Therapeutic Efficacy: A Systematic Review. Frontiers in psychiatry Frontiers Research Foundation. 2022;13:917199.
100. Ko K, Kopra EI, Cleare AJ, Rucker JJ. Psychedelic therapy for depressive symptoms: A systematic review and meta-analysis. Journal of Affective Disorders. 2023;322:194-204.
101. Kopra EI, Ferris JA, Winstock AR, Kuypers KP, Young AH, Rucker JJ. Investigation of self-treatment with lysergic acid diethylamide and psilocybin mushrooms: Findings from the Global Drug Survey 2020. Journal of Psychopharmacology. 2023:2698811231158245.
102. Kuburi S, Di Passa AM, Tassone VK, Mahmood R, Lalovic A, Ladha KS, et al. Neuroimaging Correlates of Treatment Response with Psychedelics in Major Depressive Disorder: A Systematic Review. Chronic Stress (Thousand Oaks). 2022;6:24705470221115342.
103. Kuypers KPC. The therapeutic potential of microdosing psychedelics in depression. Therapeutic Advances in Psychopharmacology. 2020;10:2045125320950567.
104. Kvam TM, Stewart LH, Andreassen OA. Psychedelic drugs in the treatment of anxiety, depression and addiction. Tidsskrift for Den Norske Laegeforening. 2018;138(18):13.
105. Lea T, Amada N, Jungaberle H, Schecke H, Scherbaum N, Klein M. Perceived outcomes of psychedelic microdosing as self-managed therapies for mental and substance use disorders. Psychopharmacology. 2020;237(5):1521-32.
106. Leger RF, Unterwald EM. Assessing the effects of methodological differences on outcomes in the use of psychedelics in the treatment of anxiety and depressive disorders: A systematic review and meta-analysis. Journal of Psychopharmacology. 2022;36(1):20-30.
107. Lehto RH, Miller M, Sender J. The Role of Psilocybin-Assisted Psychotherapy to Support Patients With Cancer: A Critical Scoping Review of the Research. Journal of Holistic Nursing. 2021:8980101211039086.
108. Li N-X, Hu Y-R, Chen W-N, Zhang B. Dose effect of psilocybin on primary and secondary depression: A preliminary systematic review and meta-analysis. Journal of Affective Disorders. 2022;296:26-34.
109. Lugo-Radillo A, Cortes-Lopez JL. Long-term amelioration of ocd symptoms in a patient with chronic consumption of psilocybin-containing mushrooms. Journal of Psychoactive Drugs. 2020:No Pagination Specified.
110. Lunsky I, Gutierrez G, Bahji A, Vazquez G. Psychedelics for the treatment of mental disorders: A systematic review and network meta-analysis. Neuropsychopharmacology. 2021;46:279.
111. Luoma JB, Chwyl C, Bathje GJ, Davis AK, Lancelotta R. A Meta-Analysis of Placebo-Controlled Trials of Psychedelic-Assisted Therapy. Journal of Psychoactive Drugs. 2020;52(4):289-99.
112. Lyes M, Yang KH, Castellanos J, Furnish T. Microdosing psilocybin for chronic pain: a case series. Pain. 2022;05:05.
113. Lyons A. Self-administration of Psilocybin in the Setting of Treatment-resistant Depression. Innov Clin Neurosci. 2022;19(7-9):44-7.
114. Lyons T, Carhart-Harris RL. More Realistic Forecasting of Future Life Events After Psilocybin for Treatment-Resistant Depression. Frontiers in Psychology. 2018;9:1721.
115. Lyons T, Carhart-Harris RL. Increased nature relatedness and decreased authoritarian political views after psilocybin for treatment-resistant depression. Journal of Psychopharmacology. 2018;32(7):811-9.
116. Machado C, Monteiro L, Fragoeiro C, Almeida B. Psilocybin: Antidepressive, anxiolytic and antiaddictive effects. European Psychiatry. 2018;48(Supplement 1):S315.
117. Madsen JD, Hoffart A. Psychotherapy with the aid of LSD. Nordic Journal of Psychiatry. 1996;50(6):477-86.
118. Madsen MK, Petersen AS, Stenbaek DS, Sorensen IM, Schionning H, Fjeld T, et al. Psilocybin-induced reduction in chronic cluster headache attack frequency correlates with changes in hypothalamic functional connectivity. medRxiv. 2022;10.
119. Maia LO, Beaussant Y, Garcia ACM. The Therapeutic Potential of Psychedelic-assisted Therapies for Symptom Control in Patients Diagnosed With Serious Illness: A Systematic Review. Journal of Pain & Symptom Management. 2022;63(6):e725-e38.
120. Malone TC, Mennenga SE, Guss J, Podrebarac SK, Owens LT, Bossis AP, et al. Individual Experiences in Four Cancer Patients Following Psilocybin-Assisted Psychotherapy. Frontiers in Pharmacology. 2018;9:256.
121. Matzopoulos R, Morlock R, Morlock A, Lerer B, Lerer L. Psychedelic mushrooms in the USA: Knowledge, patterns of use, and association with health outcomes. Frontiers in Psychiatry Vol 12 2022, ArtID 780696. 2022;12.
122. McKenna M, Fedota J, Garcia-Romeu A, Johnson M, Griffiths R, Stein E. Psilocybin improves cognitive control and downregulates parietal cortex in treatment-seeking smokers. Biological Psychiatry. 2018;83(9 Supplement 1):S231-S2.
123. Mertens LJ, Wall MB, Roseman L, Demetriou L, Nutt DJ, Carhart-Harris RL. Therapeutic mechanisms of psilocybin: Changes in amygdala and prefrontal functional connectivity during emotional processing after psilocybin for treatment-resistant depression. Journal of Psychopharmacology. 2020;34(2):167-80.
124. Monferrer M, Ricarte JJ, Montes MJ, Fernandez-Caballero A, Fernandez-Sotos P. Psychosocial remediation in depressive disorders: A systematic review. Journal of Affective Disorders. 2021;290:40-51.
125. Moreno FA, Wiegand CB, Taitano E, Delgado PL. Safety, tolerability, and efficacy of psilocybin in 9 patients with obsessive-compulsive disorder. The Journal of Clinical Psychiatry. 2006;67(11):1735-40.
126. Morton E, Michalak E, Wooley J, Pleet M, Ashtari A, Sakai K. Benefits and risks of psilocybin use in bipolar disorder: Aninternational, web-based survey. Bipolar Disorders. 2021;23(SUPPL 1):70.
127. Murphy R, Kettner H, Zeifman R, Giribaldi B, Kartner L, Martell J, et al. Therapeutic Alliance and Rapport Modulate Responses to Psilocybin Assisted Therapy for Depression. Frontiers in Pharmacology. 2021;12:788155.
128. Muttoni S, Ardissino M, John C. Classical psychedelics for the treatment of depression and anxiety: A systematic review. Journal of Affective Disorders. 2019;258:11-24.
129. Nielson EM, May DG, Forcehimes AA, Bogenschutz MP. The Psychedelic Debriefing in Alcohol Dependence Treatment: Illustrating Key Change Phenomena through Qualitative Content Analysis of Clinical Sessions. Frontiers in Pharmacology. 2018;9:132.
130. Nigam KB, Pandurangi AK. Do Hallucinogens Have a Role in the Treatment of Addictions? A Review of the Current Literature. SN Comprehensive Clinical Medicine. 2021;3(6):1385-95.
131. Noorani T, Garcia-Romeu A, Swift TC, Griffiths RR, Johnson MW. Psychedelic therapy for smoking cessation: Qualitative analysis of participant accounts. Journal of Psychopharmacology. 2018;32(7):756-69.
132. Nygart VA, Pommerencke LM, Haijen E, Kettner H, Kaelen M, Mortensen EL, et al. Antidepressant effects of a psychedelic experience in a large prospective naturalistic sample. Journal of Psychopharmacology. 2022;36(8):932-42.
133. Patchett-Marble R, O'Sullivan S, Tadwalkar S, Hapke E. Psilocybin mushrooms for psychological and existential distress: Treatment for a patient with palliative lung cancer. Canadian Family Physician. 2022;68(11):823-7.
134. Penedos S, Ramos C, Miguel M, Alves M, Paulino L, Azevedo A, et al. P.0729 Highlights of psychedelic history and current research on psilocybin application for treatment of depression - a comprehensive literature review. European Neuropsychopharmacology. 2021;53(Supplement 1):S532-S3.
135. Podrebarac SK, O'Donnell KC, Mennenga SE, Owens LT, Malone TC, Duane JH, et al. Spiritual experiences in psychedelic-assisted psychotherapy: Case reports of communion with the divine, the departed, and saints in research using psilocybin for the treatment of alcohol dependence. Spirituality in Clinical Practice. 2021;8(3):177-87.
136. Pouyan N, Halvaei Khankahdani Z, Younesi Sisi F, Lee Y, Rosenblat JD, Teopiz KM, et al. A Research Domain Criteria (RDoC)-Guided Dashboard to Review Psilocybin Target Domains: A Systematic Review. CNS Drugs. 2022;36(10):1031-47.
137. Psiuk D, Nowak E, Cholewa K, Lopuszanska U, Samardakiewicz M. The Potential Role of Serotonergic Hallucinogens in Depression Treatment. Life. 2021;11(8):29.
138. Psiuk D, Nowak EM, Dycha N, Lopuszanska U, Kurzepa J, Samardakiewicz M. Esketamine and Psilocybin-The Comparison of Two Mind-Altering Agents in Depression Treatment: Systematic Review. International Journal of Molecular Sciences. 2022;23(19):28.
139. Rabinowitz J, Lev-Ran S, Gross R. The association between naturalistic use of psychedelics and co-occurring substance use disorders. Frontiers in Psychiatry. 2023;13 (no pagination).
140. Ramachandran V, Chunharas C, Marcus Z, Furnish T, Lin A. Relief from intractable phantom pain by combining psilocybin and mirror visual-feedback (MVF). Neurocase. 2018;24(2):105-10.
141. Reiche S, Hermle L, Gutwinski S, Jungaberle H, Gasser P, Majic T. Serotonergic hallucinogens in the treatment of anxiety and depression in patients suffering from a life-threatening disease: A systematic review. Progress in Neuro-Psychopharmacology & Biological Psychiatry. 2018;81:1-10.
142. Reiff CM, Richman EE, Nemeroff CB, Carpenter LL, Widge AS, Rodriguez CI, et al. Psychedelics and psychedelic-assisted psychotherapy. American Journal of Psychiatry. 2020;177(5):391-410.
143. Rodriguez P, Barla C, Viera I, Bentancor M, Lago CP. Psilocybin for the treatment of cluster headache, case report. Cephalalgia. 2022;42(14):NP18-NP9.
144. Romeo B, Hermand M, Petillion A, Karila L, Benyamina A. Clinical and biological predictors of psychedelic response in the treatment of psychiatric and addictive disorders: A systematic review. Journal of Psychiatric Research. 2021;137:273-82.
145. Romeo B, Karila L, Martelli C, Benyamina A. Efficacy of psychedelic treatments on depressive symptoms: A meta-analysis. Journal of Psychopharmacology. 2020;34(10):1079-85.
146. Roseman L, Demetriou L, Wall MB, Nutt DJ, Carhart-Harris RL. Increased amygdala responses to emotional faces after psilocybin for treatment-resistant depression. Neuropharmacology. 2018;142:263-9.
147. Roseman L, Nutt DJ, Carhart-Harris RL. Quality of Acute Psychedelic Experience Predicts Therapeutic Efficacy of Psilocybin for Treatment-Resistant Depression. Frontiers in Pharmacology. 2017;8:974.
148. Rosenblat J, McIntyre R. Psilocybin Assisted Therapy for Treatment-Resistant Depression: A Phase II, Randomized, Feasibility Study. Neuropsychopharmacology. 2022;47(Supplement 1):203.
149. Rosenblat JD, Husain MI, Lee Y, McIntyre RS, Mansur RB, Castle D, et al. The Canadian Network for Mood and Anxiety Treatments (CANMAT) Task Force Report: Serotonergic Psychedelic Treatments for Major Depressive Disorder. Canadian Journal of Psychiatry - Revue Canadienne de Psychiatrie. 2023;68(1):5-21.
150. Ross S. Therapeutic use of classic psychedelics to treat cancer-related psychiatric distress. International Review of Psychiatry. 2018;30(4):317-30.
151. Ross S, Agin-Liebes G, Lo S, Zeifman RJ, Ghazal L, Benville J, et al. Acute and Sustained Reductions in Loss of Meaning and Suicidal Ideation Following Psilocybin-Assisted Psychotherapy for Psychiatric and Existential Distress in Life-Threatening Cancer. Acs Pharmacology & Translational Science. 2021;4(2):553-62.
152. Ross S, Bossis A, Guss J, Agin-Liebes G, Malone T, Cohen B, et al. Rapid and sustained symptom reduction following psilocybin treatment for anxiety and depression in patients with life-threatening cancer: a randomized controlled trial. Journal of Psychopharmacology. 2016;30(12):1165-80.
153. Rossi GN, Hallak JEC, Bouso Saiz JC, Dos Santos RG. Safety issues of psilocybin and LSD as potential rapid acting antidepressants and potential challenges. Expert Opinion on Drug Safety. 2022:1-16.
154. Rusanen SS, De S, Schindler EAD, Artto VA, Storvik M. Self-Reported Efficacy of Treatments in Cluster Headache: a Systematic Review of Survey Studies. Current Pain & Headache Reports. 2022;27:27.
155. Ruzickova R, Bily D, Vyhnankova M, Dubansky B, Konias V, Soucek Z. [Effect of psilocybine in chronic schizophrenias. I. Clinical findings]. Ceskoslovenska Psychiatrie. 1967;63(3):158-65.
156. Rydzynski Z, Cwynar S, Grzelak L, Jagiello W. Prelminary report on the experience with psychosomimetic drugs in the treatment of alcobolism. Activitas Nervosa Superior. 1968;10(3):273.
157. Rydzynski Z, Gruszczynski W. Treatment of alcoholism with psychotomimetic drugs. A follow-up study. Activitas Nervosa Superior. 1978;20(1):81-2.
158. Sakurai H, Yonezawa K, Tani H, Mimura M, Bauer M, Uchida H. Novel Antidepressants in the Pipeline (Phase II and III): A Systematic Review of the US Clinical Trials Registry. Pharmacopsychiatry. 2022;55(4):193-202.
159. Sarris J, McIntyre E, Camfield DA. Plant-based medicines for anxiety disorders, part 2: A review of clinical studies with supporting preclinical evidence. CNS Drugs. 2013;27(4):301-19.
160. Sarris J, Pinzon Rubiano D, Day K, Galvao-Coelho NL, Perkins D. Psychedelic medicines for mood disorders: current evidence and clinical considerations. Current Opinion in Psychiatry. 2022;35(1):22-9.
161. Schimmel N, Breeksema JJ, Smith-Apeldoorn SY, Veraart J, van den Brink W, Schoevers RA. Psychedelics for the treatment of depression, anxiety, and existential distress in patients with a terminal illness: a systematic review. Psychopharmacology. 2022;239(1):15-33.
162. Schindler EA, Gottschalk CH, Weil MJ, Shapiro RE, Wright DA, Sewell RA. Indoleamine Hallucinogens in Cluster Headache: Results of the Clusterbusters Medication Use Survey. Journal of Psychoactive Drugs. 2015;47(5):372-81.
163. Schindler EAD, Sewell RA, Gottschalk CH, Luddy C, Flynn LT, Lindsey H, et al. Exploratory Controlled Study of the Migraine-Suppressing Effects of Psilocybin. Neurotherapeutics. 2020.
164. Schindler EAD, Sewell RA, Gottschalk CH, Luddy C, Flynn LT, Zhu Y, et al. Exploratory investigation of a patient-informed low-dose psilocybin pulse regimen in the suppression of cluster headache: Results from a randomized, double-blind, placebo-controlled trial. Headache. 2022;62(10):1383-94.
165. Sevanick L. Psilocybin-assisted psychotherapy in the treatment of cancer-related psychosocial distress/anxiety. Psycho-Oncology. 2014;1):73-4.
166. Sewell R, Halpern JH, Pope HG, Jr. Response of cluster headache to psilocybin and LSD. Neurology. 2006;66(12):1920-2.
167. Shnayder S, Ameli R, Sinaii N, Berger A, Agrawal M. Psilocybin-assisted therapy improves psycho-social-spiritual well-being in cancer patients. Journal of Affective Disorders. 2023;323:592-7.
168. Shukuroglou M, Roseman L, Wall M, Nutt D, Kaelen M, Carhart-Harris R. Changes in music-evoked emotion and ventral striatal functional connectivity after psilocybin therapy for depression. Journal of Psychopharmacology. 2023;37(1):70-9.
169. Siegel AN, Meshkat S, Benitah K, Lipsitz O, Gill H, Lui LM, et al. Registered clinical studies investigating psychedelic drugs for psychiatric disorders. Journal of Psychiatric Research. 2021;139:71-81.
170. Simonsson O, Hendricks PS, Chambers R, Osika W, Goldberg SB. Classic psychedelics, health behavior, and physical health. Therapeutic Advances in Psychopharmacology. 2022;12:20451253221135363.
171. Sogaard Juul T, Ebbesen Jensen M, Fink-Jensen A. The use of classic psychedelics among adults: a Danish online survey study. Nordic Journal of Psychiatry. 2022:1-12.
172. Spriggs MJ, Giribaldi B, Lyons T, Rosas FE, Kartner LS, Buchborn T, et al. Body mass index (BMI) does not predict responses to psilocybin. Journal of Psychopharmacology. 2023;37(1):107-16.
173. Spriggs MJ, Kettner H, Carhart-Harris RL. The effect of psychedelics on mood and wellbeing in individuals reporting a diagnosis of an eating disorder. European Eating Disorders Review. 2021;29(6):E16.
174. Stauffer CS, Anderson BT, Ortigo KM, Woolley J. Psilocybin-Assisted Group Therapy and Attachment: Observed Reduction in Attachment Anxiety and Influences of Attachment Insecurity on the Psilocybin Experience. Acs Pharmacology & Translational Science. 2021;4(2):526-32.
175. Stinson S, Anderson B. (121) Treatments for the Demoralization Syndrome: A Systematic Review. Journal of the Academy of Consultation-Liaison Psychiatry. 2022;63(Supplement 2):S172.
176. Strickland JC, Garcia-Romeu A, Johnson MW. Set and Setting: A Randomized Study of Different Musical Genres in Supporting Psychedelic Therapy. ACS Pharmacol Transl Sci. 2021;4(2):472-8.
177. Stroud JB, Freeman TP, Leech R, Hindocha C, Lawn W, Nutt DJ, et al. Psilocybin with psychological support improves emotional face recognition in treatment-resistant depression. Psychopharmacology (Berl). 2018;235(2):459-66.
178. Swift TC, Belser AB, Agin-Liebes G, Devenot N, Terrana S, Friedman HL, et al. Cancer at the dinner table: Experiences of psilocybin-assisted psychotherapy for the treatment of cancer-related distress. Journal of Humanistic Psychology. 2017;57(5):488-519.
179. Trope A, Anderson BT, Hooker AR, Glick G, Stauffer C, Woolley JD. Psychedelic-Assisted Group Therapy: A Systematic Review. Journal of Psychoactive Drugs. 2019;51(2):174-88.
180. van Amsterdam J, van den Brink W. The therapeutic potential of psilocybin: a systematic review. Expert Opinion on Drug Safety. 2022:1-8.
181. van der Meer PB, Fuentes JJ, Kaptein AA, Schoones JW, de Waal MM, Goudriaan AE, et al. Therapeutic effect of psilocybin in addiction: A systematic review. Frontiers in psychiatry Frontiers Research Foundation. 2023;14:1134454.
182. Vargas AS, Luis A, Barroso M, Gallardo E, Pereira L. Psilocybin as a New Approach to Treat Depression and Anxiety in the Context of Life-Threatening Diseases-A Systematic Review and Meta-Analysis of Clinical Trials. Biomedicines. 2020;8(9):05.
183. Verroust V, Zafar R, Spriggs MJ. Psilocybin in the treatment of anorexia nervosa: The English transition of a French 1959 case study. Annales Medico-Psychologiques. 2021;179(8):777-81.
184. von Rotz R, Schindowski EM, Jungwirth J, Schuldt A, Rieser NM, Zahoranszky K, et al. Single-dose psilocybin-assisted therapy in major depressive disorder: a placebo-controlled, double-blind, randomised clinical trial. EClinicalMedicine. 2023;56.
185. Wall MB, Lam C, Ertl N, Kaelen M, Roseman L, Nutt DJ, et al. Increased low-frequency brain responses to music after psilocybin therapy for depression. bioRxiv. 2022;15.
186. Watts R, Day C, Krzanowski J, Nutt D, Carhart-Harris R. Patients' accounts of increased "connectedness" and "acceptance" after psilocybin for treatment-resistant depression. Journal of Humanistic Psychology. 2017;57(5):520-64.
187. Weissman C, Singhal N, Jones BDM, Zeifman RJ. The Placebo Response in Classic Psychedelics: A Systematic Review of Clinical Trials and Qualitative Analysis. Neuropsychopharmacology. 2022;47(Supplement 1):515.
188. Weston NM, Gibbs D, Bird CI, Daniel A, Jelen LA, Knight G, et al. Historic psychedelic drug trials and the treatment of anxiety disorders. Depression and Anxiety. 2020;37(12):1261-79.
189. White CM, Weisman N, Dalo J. Psychedelics for Patients With Cancer: A Comprehensive Literature Review. Ann Pharmacother. 2023:10600280221144055.
190. Wieckiewicz G, Stoklosa I, Piegza M, Gorczyca P, Pudlo R. Lysergic Acid Diethylamide, Psilocybin and Dimethyltryptamine in Depression Treatment: A Systematic Review. Pharmaceuticals. 2021;14(8):12.
191. Wilcox JA. Psilocybin and Obsessive Compulsive Disorder. Journal of Psychoactive Drugs. 2014;46(5):393-5.
192. Yu CL, Liang CS, Yang FC, Tu YK, Hsu CW, Carvalho AF, et al. Trajectory of Antidepressant Effects after Single- or Two-Dose Administration of Psilocybin: A Systematic Review and Multivariate Meta-Analysis. J Clin Med. 2022;11(4).
193. Yu CL, Yang FC, Yang SN, Tseng PT, Stubbs B, Yeh TC, et al. Psilocybin for End-of-Life Anxiety Symptoms: A Systematic Review and Meta-Analysis. Psychiatry Investigation. 2021;18(10):958-67.

**Ongoing studies (n=80)**

| **Title** | **URL** |
| --- | --- |
| Study of the Safety and Feasibility of Psilocybin in Adults With Methamphetamine Use Disorder | https://ClinicalTrials.gov/show/NCT05322954 |
| Psilocybin for the Treatment of Veterans With Post-Traumatic Stress Disorder | https://ClinicalTrials.gov/show/NCT05554094 |
| Psilocybin for Treatment of Alcohol Use Disorder: a Feasibility Study | https://ClinicalTrials.gov/show/NCT04718792 |
| Psilocybin-assisted CBT for Depression | https://ClinicalTrials.gov/show/NCT05227612 |
| Psilocybin-assisted Interpersonal Therapy for Depression | https://ClinicalTrials.gov/show/NCT05581797 |
| Effects of Psilocybin in Obsessive Compulsive Disorder | https://ClinicalTrials.gov/show/NCT05546658 |
| Psilocybin Therapy for Depression and Anxiety in Parkinson's Disease | https://ClinicalTrials.gov/show/NCT04932434 |
| Psilocybin in Co-occuring Major Depressive Disorder and Borderline Personality Disorder | https://ClinicalTrials.gov/show/NCT05399498 |
| Psilocybin for Major Depressive Disorder | https://ClinicalTrials.gov/show/NCT05675800 |
| Psilocybin Therapy for Depression in Bipolar II Disorder | https://ClinicalTrials.gov/show/NCT05065294 |
| A Study of Psilocybin for Major Depressive Disorder (MDD) | https://ClinicalTrials.gov/show/NCT03866174 |
| Standardized Natural Psilocybin-assisted Psychotherapy for Tapering of Opioid Medication | https://ClinicalTrials.gov/show/NCT05585229 |
| Psilocybin for Psychological and Existential Distress in Palliative Care | https://ClinicalTrials.gov/show/NCT04754061 |
| Investigating the Therapeutic Effects of Psilocybin in Treatment-Resistant Post-Traumatic Stress Disorder | https://ClinicalTrials.gov/show/NCT05243329 |
| The Safety and Efficacy of Psilocybin in Cancer Patients With Major Depressive Disorder | https://ClinicalTrials.gov/show/NCT04593563 |
| Effects of Psilocybin in Post-Treatment Lyme Disease | https://ClinicalTrials.gov/show/NCT05305105 |
| Psilocybin-Enhanced Psychotherapy for Methamphetamine Use Disorder | https://ClinicalTrials.gov/show/NCT04982796 |
| Efficacy and Safety of COMP360 Psilocybin Therapy in Anorexia Nervosa: a Proof-of-concept Study | https://ClinicalTrials.gov/show/NCT05481736 |
| Efficacy of Psilocybin in OCD: a Double-Blind, Placebo-Controlled Study. | https://ClinicalTrials.gov/show/NCT03356483 |
| The Effects of Psilocybin on Self-Focus and Self-Related Processing in Treatment Resistant MDD | https://ClinicalTrials.gov/show/NCT05381974 |
| Frontline Clinician Psilocybin Study | https://ClinicalTrials.gov/show/NCT05163496 |
| Psilocybin for Treatment-Resistant Depression | https://ClinicalTrials.gov/show/NCT05029466 |
| Psilocybin-assisted Therapy for Treatment of Alcohol Use Disorder | https://ClinicalTrials.gov/show/NCT05416229 |
| Effects of Psilocybin in Anorexia Nervosa | https://ClinicalTrials.gov/show/NCT04052568 |
| Psilocybin for Treatment of Obsessive Compulsive Disorder | https://ClinicalTrials.gov/show/NCT03300947 |
| Psilocybin for Depression in People With Mild Cognitive Impaircasement or Early Alzheimer's Disease | https://ClinicalTrials.gov/show/NCT04123314 |
| Effects of Repeated Psilocybin Dosing in OCD | https://ClinicalTrials.gov/show/NCT05370911 |
| The Safety and Efficacy of Psilocybin in Patients With Treatment-resistant Depression and Chronic Suicidal Ideation | https://ClinicalTrials.gov/show/NCT05220410 |
| Psilocybin in Functional Neurological Disorder | https://ClinicalTrials.gov/show/NCT05723276 |
| Effect of SSRIs on Response to Psilocybin Therapy | https://ClinicalTrials.gov/show/NCT05594667 |
| Does Psilocybin Require Psychedelic Effects to Treat Depression? | https://ClinicalTrials.gov/show/NCT05710237 |
| Investigating the Mechanisms of the Effects of Psilocybin on Visual Perception and Visual Representations in the Brain | https://ClinicalTrials.gov/show/NCT05265546 |
| Psilocybin-Assisted vs Ketamine-Assisted Psychotherapy for Alcohol Use Disorder | https://ClinicalTrials.gov/show/NCT05421065 |
| Psilocybin as a Treatment for Anorexia Nervosa: A Pilot Study | https://ClinicalTrials.gov/show/NCT04505189 |
| Psilocybin - Induced Neuroplasticity in the Treatment of Major Depressive Disorder | https://ClinicalTrials.gov/show/NCT03554174 |
| Psilocybin-facilitated Treatment for Chronic Pain | https://ClinicalTrials.gov/show/NCT05068791 |
| Psilocybin-Assisted Psychotherapy in Adults With Alcohol Use Disorder (AUD) | https://ClinicalTrials.gov/show/NCT05646303 |
| Exploratory Study of Low Dose Psilocybin | https://ClinicalTrials.gov/show/NCT05227742 |
| Psilocybin-assisted Therapy for Phantom Limb Pain | https://ClinicalTrials.gov/show/NCT05224336 |
| Psilocybin for the Treatment of Migraine Headache | https://ClinicalTrials.gov/show/NCT03341689 |
| A Phase II, Multicentre, Randomised, Double-blind, Controlled Study to Investigate the Safety, Tolerability, Pharmacokinetics, and Efficacy of COMP360 in Participants With Major Depressive Disorder With One Prior Treatment Failure | https://ClinicalTrials.gov/show/NCT05733546 |
| Psilocybin in Adults With and Without Autism Spectrum Disorder | https://ClinicalTrials.gov/show/NCT05651126 |
| Psilocybin for Opioid Use Disorder in Patients on Methadone Maintenance With Ongoing Opioid Use | https://ClinicalTrials.gov/show/NCT05242029 |
| Repeat Dosing of Psilocybin in Migraine Headache | https://ClinicalTrials.gov/show/NCT04218539 |
| The Safety and Efficacy of Psilocybin in Participants With Type 2 Bipolar Disorder (BP-II) Depression. | https://ClinicalTrials.gov/show/NCT04433845 |
| An Open Label Study of the Safety and Efficacy of Psilocybin in Participants With Treatment-Resistant Depression (P-TRD) | https://ClinicalTrials.gov/show/NCT04433858 |
| 5-HT2A Agonist Psilocybin in the Treatment of Tobacco Use Disorder | https://ClinicalTrials.gov/show/NCT05452772 |
| Safety and Tolerability of Psilocybin in Post-Traumatic Stress Disorder | https://ClinicalTrials.gov/show/NCT05562973 |
| Pilot Study of Psilocybin-Assisted Therapy for Demoralization in Patients Receiving Hospice Care | https://ClinicalTrials.gov/show/NCT04950608 |
| Study of Psilocybin Enhanced Group Psychotherapy in Patients With Cancer | https://ClinicalTrials.gov/show/NCT04522804 |
| Psilocybin Versus Ketamine in Treatment-Resistant Depression | https://ClinicalTrials.gov/show/NCT05383313 |
| Clinical and Mechanistic Effects of Psilocybin in Alcohol Addicted Patients | https://ClinicalTrials.gov/show/NCT04141501 |
| Psilocybin-facilitated Treatment for Cocaine Use | https://ClinicalTrials.gov/show/NCT02037126 |
| Effects of Psilocybin in Concussion Headache | https://ClinicalTrials.gov/show/NCT03806985 |
| Psilocybin Treatment of Major Depressive Disorder With Co-occurring Alcohol Use Disorder | https://ClinicalTrials.gov/show/NCT04620759 |
| Adjunctive Effects of Psilocybin and a Formulation of Buprenorphine | https://ClinicalTrials.gov/show/NCT04161066 |
| Pilot Trial of Visual Healing® in Psilocybin-assisted Therapy for Alcohol Use Disorder | https://ClinicalTrials.gov/show/NCT04410913 |
| Pragmatic Trial of Psilocybin Therapy in Palliative Care | https://ClinicalTrials.gov/show/NCT05403086 |
| Psilocybin-facilitated Smoking Cessation Treatment: A Pilot Study | https://ClinicalTrials.gov/show/NCT01943994 |
| Evaluating the Feasibility, Safety and Efficacy of Psychotherapy Assisted Psilocybin for Treatment of Severe OCD | https://ClinicalTrials.gov/show/NCT04882839 |
| Psilocybin Combined With Multidisciplinary Palliative Care in Demoralized Cancer Survivors With Chronic Pain | https://ClinicalTrials.gov/show/NCT05506982 |
| Psilocybin Therapy for Chronic Low Back Pain | https://ClinicalTrials.gov/show/NCT05351541 |
| Psilocybin Therapy in Advanced Cancer | https://ClinicalTrials.gov/show/NCT05398484 |
| Northwest Therapies Trauma Psilocybin Study Compassionate Use Study | https://ClinicalTrials.gov/show/NCT05042466 |
| The Effect of Psilocybin on MDD Symptom Severity and Synaptic Density | https://ClinicalTrials.gov/show/NCT04630964 |
| Psilocybin in Depression Resistant to Standard Treatments | https://ClinicalTrials.gov/show/NCT04959253 |
| Psilocybin in Patients With Fibromyalgia: EEG-measured Brain Biomarkers of Action | https://ClinicalTrials.gov/show/NCT05548075 |
| Efficacy and Safety of Psilocybin in Treatment-Resistant Major Depression | https://ClinicalTrials.gov/show/NCT04670081 |
| Psilocybin for the Treatment of Cluster Headache | https://ClinicalTrials.gov/show/NCT02981173 |
| PAPR: PAP + MBSR for Front-line Healthcare Provider COVID-19 Related Burnout | https://ClinicalTrials.gov/show/NCT05557643 |
| Evaluation of Psilocybin (TRP-8802) in the Treatment of Binge Eating Disorder | https://ClinicalTrials.gov/show/NCT05035927 |
| Efficacy, Safety, and Tolerability of Two Administrations of COMP360 in Participants With TRD | https://ClinicalTrials.gov/show/NCT05711940 |
| Palliadelic Treatment to Reduce Psychological Distress in Persons With Inoperable Pancreatobiliary Cancer | https://ClinicalTrials.gov/show/NCT05220046 |
| Open-label Study to Assess the Safety and Efficacy of TRP-8802 With Psychotherapy in Adult Participants With Fibromyalgia | https://ClinicalTrials.gov/show/NCT05128162 |
| Microdosing Psychedelics to Improve Mood | https://ClinicalTrials.gov/show/NCT05259943 |
| The Safety and Tolerability of COMP360 in Participants With Post-traumatic Stress Disorder | https://ClinicalTrials.gov/show/NCT05312151 |
| Efficacy, Safety, and Tolerability of a Single Administration of COMP360 in Participants With TRD | https://ClinicalTrials.gov/show/NCT05624268 |
| A Study of a Psilocybin Analog (CYB003) in Healthy Participants With and Without Major Depressive Disorder | https://ClinicalTrials.gov/show/NCT05385783 |
| ELE-101 Safety & Tolerability Study in Healthy Participants and Patients With Depression | https://ClinicalTrials.gov/show/NCT05434156 |
| Ketamine-Assisted PsychoTherapy ViAbility in Treating Cancer-related Emotional Distress | https://ClinicalTrials.gov/show/NCT05344625 |
